# Infectious Complications in Autoimmune Hemolytic Anemia

**DOI:** 10.3390/jcm10010164

**Published:** 2021-01-05

**Authors:** Juri Alessandro Giannotta, Bruno Fattizzo, Francesca Cavallaro, Wilma Barcellini

**Affiliations:** 1 Hematology Unit, Fondazione IRCCS Ca’ Granda Ospedale Maggiore Policlinico, Via Francesco Sforza 35, 20100 Milan, Italy; bruno.fattizzo@unimi.it (B.F.); francesca.cavallaro@unimi.it (F.C.); wilma.barcellini@policlinico.mi.it (W.B.); 2 Department of Oncology and Oncohematology, University of Milan, Via Festa del Perdono 7, 20100 Milan, Italy

**Keywords:** autoimmune hemolytic anemia, steroids, rituximab, immunosuppressants, prophylaxis, viral reactivation

## Abstract

Autoimmune hemolytic anemia (AIHA) may be frequently challenged by infectious complications, mainly as a result of immunosuppressive treatments administered. Furthermore, infectious agents are known triggers of AIHA onset and relapse. Although being risk factors for mortality, infections are an underestimated issue in AIHA. This review will collect the available evidence on the frequency and type of infectious complications in AIHA, detailing the risk related to each treatment (i.e., steroids, rituximab, splenectomy, classic immunosuppressive agents, and new target drugs). Moreover, we will briefly discuss the infectious complications in AIHA secondary to other diseases that harbor an intrinsic infectious risk (e.g., primary immunodeficiencies, systemic autoimmune diseases, lymphoproliferative disorders, solid organ and hematopoietic stem cell transplants). Finally, viral and bacterial reactivations during immune suppressive therapies will be discussed, along with suggested screening and prophylactic strategies.

## 1. Introduction

Autoimmune hemolytic anemia (AIHA) encompasses a group of heterogeneous conditions mainly characterized by red blood cell (RBC) lysis due to autoantibodies against surface erythrocyte’s antigens. Based on the thermal characteristics of the autoantibody, AIHAs can be classified into warm forms, generally caused by IgG antibodies reacting at warm temperatures and able to fix complement in some cases; cold agglutinin disease (CAD), due to IgM antibodies that agglutinate RBCs at low temperatures and lyse them via the complement cascade activation; and mixed forms (coexistence of warm and cold autoantibodies) [1,2]. Infections in AIHA are a known player in the pathogenesis of the autoimmune process. On the other hand, infections can occur also as consequence of the disease and its treatments. There is increasing awareness of infections in AIHA, as they can impact on outcome, including morbidity and fatality. Additionally, AIHA can be secondary to systemic autoimmune diseases and lymphoproliferative disorders, whose treatments may further increase the infectious risk. Likewise, AIHA is frequently observed in primary immunodeficiencies (PIDs) that are characterized by a well-known infectious diathesis. The clinical management of infections and prophylactic measures in primary AIHA remains largely unknown, at variance with secondary forms. The only available data derive mainly from retrospective series and case reports, or from more recent clinical trials with novel drugs.

In this review we will focus on the frequency and type of infectious complications in primary and secondary AIHA. We will discuss the infectious risk related to each treatment, including new target drugs, and the issue of viral and mycobacterial reactivations during immunosuppressive therapies. Screening and prophylactic strategies will be also provided.

## 2. Prevalence of Infections in Primary AIHA

In a large multicenter Italian study on 308 primary AIHAs, infectious complications were registered in 26 patients (8.4%), of whom 11 were grade 4 (according to the Common Terminology Criteria for Adverse Events) and five were fatal [3]. They consisted mostly in pneumonia, and the causative agents were *Pneumocystis jirovecii*, Mycoplasma pneumoniae, Staphylococcus epidermidis, Pseudomonas aeruginosa, Varicella Zoster virus (VZV), and Candida albicans. They were not associated with AIHA type and severity nor with the number of medical therapies. Notably, splenectomy was the only factor associated with severe infectious complications (grade ≥ 3). The same authors in a subsequent larger series with an extended follow-up reported a higher proportion of infections (14%), mostly in warm and mixed subtypes [4]. In another study on 33 primary AIHA patients from South India followed for a median period of 50 months, four developed infective episodes (two pneumonias, one popliteal abscess, and one sepsis, with no isolations): one was grade 2, two were grade 4, and one resulted in a fatal sepsis. In detail, one pneumonia occurred on a very low dose of prednisone (<5 mg/day) and azathioprine (AZA), while the other complicated a cerebral vein thrombosis requiring intensive care [5]. More recently, in an Italian single-center analysis of 225 primary AIHAs, a total of 45 infections were recorded in 29 patients (up to four episodes in one patient) over a 3-year follow-up. Two third of infections were >G3 and two were fatal (post-splenectomy sepsis and Pneumocystis jiroveci pneumonia). Of note, 60% occurred during an active phase of the disease and 14% at the time of AIHA diagnosis. Patients with infectious complications also had higher rates of thrombotic events and Evans’ syndrome (association with immune thrombocytopenia). Additionally, patients with infections had received more lines of therapy, particularly rituximab, splenectomy, and immunosuppressants (AZA, cyclophosphamide and cyclosporine) [6]. Focusing on fatal outcome, a study of 83 AIHA patients in the period 1980–2000 reported 13 deaths, of which five related to infective episodes [7]. A single-center French experience on 60 warm AIHAs reported two deaths, both occurring in splenectomized patients, and still on immunosuppressive therapy. In detail, the first patient had superimposed pneumococcal-related sepsis complicating a H1N1 influenza pneumonia, and the second a Gram-negative bacilli-related sepsis in a previous history of pneumocystosis and pulmonary aspergillosis [8]. In the Italian study [3], the occurrence of infections was strongly associated with death (hazard ratio 11.47; 95% CI 3.43–38.4, *p* = 0.0004). Finally, a study of 101 warm AIHA performed in Thailand indicated sepsis as the most common cause of death, supervened in 11% of patients [9]. Altogether these findings indicate that infections in AIHA occur in 6–14% of cases, and may result in fatal outcome.

## 3. Infectious Risk Associated with AIHA Therapies

The risk and type of infections associated with AIHA treatments differ according to the dose, the time of exposure and the depth of immunosuppression induced by each therapy. Table 1 summarizes the main findings for the different AIHA treatments, detailed separately as follows.

### 3.1. Steroids

Steroid-associated infectious risk has been largely reported, together with other side effects (i.e., osteoporosis, diabetes mellitus, and hypertension). The mechanisms by which corticosteroids impair the immune response against pathogens are multiple, including reduced opsonization and phagocytosis of bacteria, impaired T cell function (increasing the risk for mycobacterial, viral, and fungal infection), and enhanced eosinophil apoptosis (favoring parasitic infections). In fact, several opportunistic infections have been reported, i.e., *Pneumocystis jirovecii* pneumonia (PJP; especially with doses >30 mg/day) [31], aspergillosis, candidiasis, strongyloidiasis, cryptococcosis, and VZV and tuberculosis (TB) reactivations [32]. A recent large study (more than 275,000 adults with various conditions) reported a significantly higher risk of infections in the steroid-exposed group. In detail, hazard risk ranged from 2.01 for cutaneous cellulitis to 5.84 for lower respiratory tract infections, and it correlated positively with steroid dose, independently of the underlying condition [10]. In rheumatoid arthritis (RA), the increased risk of infections was attributable to steroids even at low doses (i.e., 5 mg/day or less) [33]. Moreover, a case–control study conducted on almost 12,000 over-65 RA patients found that continuous treatment with 5 mg prednisolone for the last 3 months, 6 months, or 3 years had a 30%, 46%, or 100% increased risk of serious infections, respectively, halving the risk only many months after discontinuation [11]. Similarly, in systemic lupus erythematosus (SLE) infections are one of the leading causes of morbidity and mortality, and a dose >7.5–10 mg/day of prednisone is a well-recognized risk factor [34]. Although being the backbone therapy, no studies addressed the steroid-related infectious risk in AIHA. Retrospective data showed that primary AIHA patients experiencing infections received a mean cumulative dose of corticosteroids of 8.2 kg for a median time of 12 months before the first event [6]. Finally, it is noteworthy the description of six cases of cryptococcal infections in AIHA treated with steroids only. One was an elderly non-HIV non-transplanted patient who developed disseminated cryptococcal disease while receiving high-dose prednisone (100 mg/day) [35]; the other five AIHA patients contracted cryptococcal meningitis while on prednisone >15 mg/day [12]. Altogether, these data suggest that steroids represent a risk factor for infections, particularly at high doses but also at low and prolonged regimens. Moreover, they are also associated with infections caused by uncommon agents, including fungi and parasites.

### 3.2. Rituximab

Rituximab is a chimeric anti-CD20 monoclonal antibody targeting B cells, used as single agent or combined with chemotherapy. It has proven effective both in warm and cold AIHAs, representing the preferred option at relapse in the former and the first-line treatment in the latter [1,2,13]. Rituximab has been associated with an increased infectious risk, related to its B cell and immunoglobulin-depleting effect. A clear association is established between rituximab and progressive multifocal leukoencephalopathy (PML), mostly in hematologic malignancies and bone marrow transplantations [14]. PML is a devastating demyelinating disease of central nervous system caused by the reactivation of John Cunningham virus (JCV), a polyomavirus that latently infects the kidneys of almost 50% of healthy adults. More uncommon infections related to rituximab are described in retrospective case series, and include PJP, enterovirus encephalitis, parvovirus B19, cytomegalovirus (CMV), West Nile virus, and babesiosis [36]. In follicular lymphoma, a meta-analysis shows that severe infections occurred when the drug is used as maintenance therapy [37]. At variance with lymphoproliferative disorders, data on systemic autoimmune disorders indicate that rituximab is not associated with a significant infectious risk. In fact, PML is only a rare complication, reported to be less than 2/100,000 patients in systemic vasculitides [38]. Moreover, only mild infections are reported in RA clinical trials [39]. Finally, a recent systematic review evaluating rituximab use in autoimmune diseases found no difference in infectious rates between rituximab- vs. non-rituximab-treated patients [15].

As regards AIHA, a meta-analysis including 21 studies reported an incidence of about 5% of severe infections, including one PJP [16]. A similar incidence was found in a French retrospective study of autoimmune cytopenias associated with SLE. In this cohort severe non-opportunistic infections occurred in 4.2% patients, with an estimated incidence of 1.2 severe infections/100 patient-years [40]. In cold AIHA, rituximab showed a good safety profile, with only 3% of G1 infections reported, although one fatal pneumonia 9 months after the end of therapy was recorded [41]. The same good safety profile has been reported in a cohort of elderly AIHA patients, in which only two urinary tract infections were registered over a median 31-month follow-up period [42]. As regards the low-dose rituximab regimen (i.e., 100 mg i.v. weekly for 4 weeks), no infections were registered in a median follow-up of 15 months (range 6–35) [43]. Surprisingly, the only study reporting a higher incidence of infections is an Asian case–control study in which the infectious rate was about 35% with low-dose rituximab, comparable to the cyclophosphamide (CTX)-treated arm [44]. Infection rates rise when rituximab is combined to chemotherapy. In perspective studies, Berentsen et al. reported an infection rate of 11% for bendamustine association and up to 59% when associated to fludarabine, including two fatal pneumonias [45,46]. Finally, no published data exist about PML incidence in AIHA, although this complication should be always considered in immunocompromised subjects. Taken together, these results suggest that rituximab as single agent is safe in AIHA, although associations with chemotherapy deserve higher attention.

### 3.3. Splenectomy

Splenectomy shows response rates similar to rituximab in warm AIHA, although long-term outcomes are poorly known; it is not effective in CAD, where extravascular hemolysis occurs mainly in the liver [1,2]. Splenectomy exposes patients to an increased risk of infections along with thrombotic events. For all these reasons, it is usually deferred after other second-line medical therapies; nonetheless, it still represents an option in multi-refractory warm AIHAs. A literature review for the period 1966–1996, including 6942 splenectomised patients for different reasons, found an infectious crude rate of 3% [47]. More recently, in a Danish nationwide analysis of about 4000 splenectomised patients [48] the overall incidence of infections was 7.7/100 patient-years vs. 2/100 for the general population. Finally, a multicenter analysis of 233 splenectomised immune thrombocytopenic (ITP) patients [49] reported a total number of 159 infections (two of them fatal) in 31% of patients. The main threat in asplenic patients consists of encapsulated bacteria, whose phagocytosis is impaired in the absence of splenic macrophages. In a large study including 349 septic episodes in asplenic patients, Streptococcus pneumonia was responsible for 57% of infections and 59% of deaths; Haemophilus influenzae for 6% of infections, with a mortality rate of 32%; and Neisseria meningitidis caused 3.7% of events [18]. A particularly harmful event is overwhelming post-splenectomy infection (OPSI), i.e., a fulminating sepsis, meningitis, or pneumonia caused by encapsulated bacteria. It occurs more commonly within the first two years (range: 1 week to 20 years) and may rapidly evolve in few hours to death, if not adequately and timely treated. The mortality rate ranges from 10 to 70%, despite adequate treatment [50]. OPSI incidence and mortality greatly depend on age (more common in children <2 years old) and on the underlying disease, being higher in hematological disorders [18]. Finally, malaria and babesiosis may be more severe in asplenic patients, who lack the physiologic filter of the infected erythrocytes [18]. Other microorganisms reported include Ehrlichia, Bacteroides, Enterococcus, Salmonella, and Bartonella [51]. The risk was generally higher in children and hereditary anemias, namely, thalassemia and sickle cell disease, while the lowest risk was observed in ITP patients.

Concerning AIHA, a systematic review including four studies and 48 splenectomised patients, found a post-operative infection rate of 6%, although data of long-term follow-up were missing [52]. Similarly, a more recent study on more than 4500 AIHA patients reported an incidence of 6.7% of sepsis in splenectomised subjects, including the late post-operative period [19]. Finally, some authors indicate splenectomy as a safe option if infections are adequately and promptly treated. In fact, in a series of 255 hematologic patients no cases of splenectomy-related sepsis occurred during a median follow-up of 35 months [53].

### 3.4. Immunosuppressive Agents

Immunosuppressive drugs are all associated with an intrinsic infectious risk, generally attributable to bone marrow toxicity. A systematic review and network metanalysis in lupus nephritis, including a total of 32 randomized clinical trials with 2611 patients, found that CTX, both low- and high-dose, mycophenolate mofetil (MMF), and AZA were associated with significantly higher risk compared to tacrolimus [20]. For CTX, an incidence of infections (bacterial, fungal, viral, protozoal, and parasitic) ranging from 15 to 34% has been described [9,54]. Notably, AZA and MMF have been associated with atypical pathogens like Listeria monocytogenes and Mycobacterium species [55,56], fungal (*Cryptococcus neoformans*, *Aspergillus*, *Mucor*, and *Pneumocystis jirovecii*) and parasitic infections (Toxoplasma gondii) [57,58]. Moreover, polyomavirus (BK virus and JCV) infections have been reported with these two drugs [9]. Conversely, the incidence of infections in autoimmune patients treated with cyclosporine A (CSA) is reported as low as 1% in clinical trials, and viral reactivation are rare [21]. Regarding AIHA, CTX toxicity is well described, also at low doses (1–2 mg/kg/day) [22], with bacterial pneumonia being the most common infection. The infectious risk related to CSA, MMF, and AZA in AIHA is less known since their use in this setting is described mostly as case series [59,60,61]. Taken together, these data indicate that treatment with classic immunosuppressants, especially CTX, is burdened by a relevant infectious risk, often characterized by atypical and opportunistic pathogens.

### 3.5. New Target Drugs

The progressive availability of new target therapies has involved also AIHA in the last years. Their infectious risk is less clear, and data derive mainly from use in diseases other than AIHA.

Upstream complement inhibitors, targeting C1s, C1q, and C3, are under investigation in cold and warm AIHAs, and the C5-inhibitor eculizumab has been used with some efficacy in CAD [23]. These drugs cause an increased susceptibility to infections, due to the impaired opsonisation and lysis of capsulated microorganisms. Particularly, in eculizumab-treated patients with paroxysmal nocturnal hemoglobinuria there is a warning for Neisseria meningitidis infections. Data from 10-year pharmacovigilance reported 76 cases of meningococcal infections (0.25/100 patient-years), eight of which fatal. With the strict adoption of vaccination policies, the meningococcal infection rate has been decreasing over time, but mortality remains considerable [24]. In addition, a recent study demonstrated that continuous C5 blocking impairs IgG-mediated complement activation, suggesting that even patients receiving adequate vaccinations against Neisseria meningitidis may not be sufficiently protected [62]. Eculizumab has been also rarely associated with pneumonia, cellulitis, bacteremia, and urinary tract infections, due to Staphylococcus, Klebsiella oxytoca, Escherichia hermannii, viruses, and fungi [24,63]. The infectious risk associated with new complement inhibitors appears very low [64], most probably due to the extended vaccination policies required for enrollment. As a general comment, C1s- and C1q-inhibitors block only the classical complement pathway, leaving the alterative and the lectin ones intact [23], while C3 inhibition may impair complement activity more profoundly.

A new treatment option for AIHA is targeting the B cell receptor signaling with drugs successfully used in chronic lymphocytic leukemia (CLL) and other lymphoproliferative disorders, such as Bruton tyrosine kinase (BTK) and phosphatidylinositol 3-kinase delta (PI3Kδ) inhibitors [65,66]. Ibrutinib use in lymphoproliferative diseases is associated with increased risk of bacterial and fungal infections, up to 40% in clinical trials and real-life experience [25,67]. Parsaclisib, a next-generation and highly selective PI3Kδ inhibitor, has shown to be effective in a phase 1–2 trial in relapsed/refractory B-cell malignancies, and a clinical trial in AIHA is ongoing at the time of writing (NCT03538041). It has the same mechanism of action as idelalisib, which has been associated with severe infectious complications in CLL patients, particularly PJP [25]. However, only three septic episodes in a cohort of 72 lymphoma patients treated with parsaclisib have been registered [68]. Fostamatinib, a spleen tyrosine kinase inhibitor proven effective in RA and ITP, is under study in relapsed AIHA (NCT03764618). In a meta-analysis of patients with RA a 20% increase in infectious risk has been reported [26]. Conversely, studies in ITP patients did not report infectious events [69].

Proteasome inhibitors such as bortezomib have also been used in AIHA with a good safety profile [27,28], at variance with the warnings reported for multiple myeloma [70].

Finally, targeting the neonatal Fc receptor (FcRn) is showing promising results in autoantibody-mediated diseases, including ITP [29,30]. FcRn rescues immunoglobulins (Ig) G from lysosomal degradation, prolonging antibody’s (and autoantibody’s) half-life. Its inhibition has a therapeutic effect by reducing the pathogenic autoantibodies. However, it causes also the reduction of other protective immunoglobulins, resulting in hypogammaglobulinemia, although not associated with clinically relevant infections [29].

Taken together, data about new target therapies in AIHA show an overall good safety profile, even though each drug carries a specific spectrum of possible related infections.

## 4. Viral and Mycobacterial Reactivations during AIHA Treatments

### 4.1. Hepatotropic Viruses

Hepatitis B virus (HBV) reactivation is defined as a sudden significant increase (>100-fold) in HBV-DNA levels in subjects with previously detectable HBV-DNA or reappearance of viral DNA in those who did not have viremia prior to the initiation of an immunosuppressive therapy. The increased viral replication can lead to hepatic damage, especially if immunosuppression is abruptly stopped, causing an immune reconstitution inflammatory response. HBV reactivation is reported in 10–40% of patients receiving chemotherapy or immunosuppressants for solid tumors and oncohematologic diseases [17]. In autoimmune disorders the rate is lower, as reported in a recent Italian survey on almost 1000 rheumatologic patients [71]. No cases have been reported in AIHA, although HBV reactivation has been described with steroids, rituximab and immunosuppressants [17]. Reactivation can be related to host (higher in males, elderly, in presence of cirrhosis), viral status (high baseline HBV-DNA, HBeAg positivity, chronic hepatitis B with HbsAg positivity) and type/degree of immunosuppression [72]. More precisely, it can be stratified into high (≥10%), moderate (1–10%) and low (<1%) risk according to the type of immunosuppressant and the HBsAg/HBcAb status [17] (Table 2). B-cell depleting agents like rituximab are at high risk of reactivation, irrespective of HbsAg/HBcAb status. Time to reactivation is highly variable and ranged from 0 to 12 months (median 3 months) in a meta-analysis on 183 lymphoma patients [73]. Contrarily, steroids are classified in different risk groups, according to the viral status and dose.

Hepatitis C virus (HCV) reactivation is rarer than HBV, although its morbidity and mortality rates are not inferior [74]. It has been described anecdotally in rheumatic and lymphoma patients receiving rituximab or chemotherapies [75,76]. In the past, HCV-positive patients displayed a higher rate of severe hepatotoxicity in rituximab-containing regimens for lymphomas [77]. Nowadays, HCV reactivation is becoming less concerning, as treatment of HCV is highly effective (cure rates approaching 100% in adherent patients).

### 4.2. Herpesviruses

Herpesvirus reactivations are frequent after solid organ or allogeneic stem cell transplants. CMV reactivation occurs in 12–67% of patients undergoing autologous stem cell transplant and 2–39% oncohematologic non-transplant subjects [78]. A clear association with alemtuzumab emerged from prospective studies [79]. Additionally, CMV and Epstein–Barr virus (EBV) reactivations have been largely described in aplastic anemia patients treated with anti-thymocyte globulin and CSA [80,81]. This indicates that an impaired T cell immunity may represent an important risk factor. Other drugs possibly associated with CMV reactivation are high-dose steroids (i.e., prednisone >1 mg/kg/day), rituximab, bortezomib, bendamustine, and fludarabine [82]. Of note, idelalisib and ibrutinib have been related to life-threatening CMV infections [83], recommending laboratory monitoring for CMV-DNA during treatment with the former.

In the context of AIHA, herpesvirus reactivations have been described only sporadically. A fulminant case of multiple organ dysfunction caused by VZV reactivation has been reported in an 80-year-old patient treated with prednisone 1 mg/kg [84]. Moreover, a fatal case of viral hepatitis (diagnosis on liver biopsy) in a CLL complicated by AIHA requiring steroids has been described [85]. EBV reactivation has been reported in an 88-year-old man treated with low-dose prednisolone [86]. Finally, a case of CMV reactivation after low-dose steroids in a 21-year-old immunocompetent AIHA has been described [87].

Taken together, these data indicate that herpesvirus reactivations represent a serious issue in older, immunocompromised patients, especially those undergoing T-cell directed therapies. In addition, it is advisable to pay attention to high-dose steroids, rituximab in combination with chemotherapies, and other B cell target therapies.

### 4.3. Tuberculosis

Host responses against *Mycobacterium tuberculosis* are mediated by a delicate interplay between innate and adaptive immunity, dominated by macrophages and T cells, respectively. An alteration of these regulatory mechanisms may result in active TB infection/reactivation [88]. In RA, glucocorticoids and methotrexate are associated with a slightly increased risk of TB infection, whilst tumor necrosis factor (TNF)-inhibitors carry a 4- to 8-fold risk [89]. A case-control study indicates an adjusted odds ratio for tuberculosis of 2.8 in patients receiving <15 mg/day prednisone-equivalent doses. The risk increases to 7.7-fold for higher doses, particularly for intravenous pulse methylprednisolone [90,91]. B cells appear to play a minor role in controlling TB infection [92]. Consistently, rituximab has not been associated with TB reactivation in patients with RA, Sjogren’s syndrome, SLE, mixed cryoglobulinemia, and vasculitides [88]. In AIHA, the risk of TB reactivation seems low, although attention should be paid to high and/or prolonged steroids use.

## 5. Prevention Strategies

Screening tests and prophylactic strategies before a specific AIHA treatment are lacking, and most indications derive from other diseases (Table 3).

### 5.1. HBV and HCV

HBV screening is indicated before any chemotherapy or immunosuppressive therapy, particularly before rituximab, according to the European Association for the Study of the Liver (EASL) and the Center for Disease Control and Prevention (CDC) guidelines [93,103]. HCV screening is also advisable before immunosuppression, particularly in patients at risk (substance users, tattoos, hemodialysis, transfusions/surgery before 1994, born from HCV-positive mother, HIV-positive, elevated liver enzymes/liver disease) [9,95]. HBV/HCV testing should be preferably performed before or 3 months after immunoglobulin administration, because of possible false positivity derived from passive transmission of anti-HBc antibodies [104]. Concerning HBV prophylaxis (Table 4), the recommended antiviral strategies vary across risk categories (previously detailed) and according different guidelines [93,94] (EASL and American Gastroenterological Association, AGA). The main discrepancy is for the moderate risk category, where AGA suggests active prophylaxis, whilst EASL indicates preemptive therapy (HBsAg and/or HBV-DNA monitoring during and after immunosuppression, starting antiviral therapy in case of positivity) [93,105]. Finally, some authors also consider HBeAg/Ab status in HBsAg-negative patients, suggesting entecavir or tenofovir in case of HBeAg positivity [106]. As a general rule, prophylaxis should continue for at least 18 months after the end of immunosuppressive therapy, monitoring serology/DNA levels for at least 12 months after prophylaxis withdrawal. Of note, EASL guidelines recommend vaccination for HBV seronegative patients who are candidate for immunosuppressive treatments [93].

### 5.2. Tuberculosis

Screening for TB is a well-defined issue in rheumatologic patients undergoing immunosuppressive therapy with anti-TNF agents, with consequent prophylaxis in latent infections. Tuberculin skin test or serum interferon release assays are recommended, with an additional chest radiograph to exclude active TB in positive subjects [107]. Given the described risk of TB reactivation under long-term steroid therapy, the CDC guidelines recommend following the above-mentioned strategies for long-term prednisone users [9,96].

### 5.3. Pneumocystis jirovecii

PJP is life-threatening in immunocompromised patients. Adequate antimicrobial prophylaxis is mandatory in conditions like allogeneic stem cell transplants, high-dose chemotherapy regimens and patients receiving long-term steroids for hematological malignancies [97]. The mortality of PJP is quite high also in patients with autoimmune diseases, reaching even 50% in some settings [108,109]. In AIHA and other autoimmune disorders, PJP prophylaxis is not defined, and some experts suggest it in patients receiving >10–20 mg/day prednisone-equivalent doses for more than 4 weeks with additional risk factors (age >65, pre-existing pulmonary disease, combination therapy with CTX or rituximab) [9,98]. Additionally, PJP prophylaxis is requested in the clinical trial with the PI3Kd inhibitor parsaclisib, due to the warning of PJP observed with idelalisib (NCT03538041).

### 5.4. Herpesvirus Reactivations

Clear evidence supports antiviral prophylaxis in transplanted patients or those receiving high-dose chemotherapy [110,111]. The German Society for Hematology and Medical Oncology suggests (val)acyclovir for herpes simplex virus and VZV in patients treated with alemtuzumab, bortezomib, or purine analogs [112]. The European Conference on Infections in Leukaemia Group recommends monitoring of CMV in transplanted patients to promptly start pre-emptive antiviral therapy [113]. No clear evidence exists about herpesvirus prophylaxis in autoimmune diseases, nor in AIHA.

### 5.5. Vaccinations

For patients undergoing elective splenectomy, vaccination against encapsulated bacteria is recommended. Moreover, influenza virus (to be repeated annually) and VZV vaccines in over 50-year-old subjects are also advised [99,100,101,102]. Vaccines against encapsulated bacteria include the 23-valent polysaccharide pneumococcal vaccine, which covers around 70–90% of strains [18], the 13-valent conjugate pneumococcal, meningococcal vaccines against ACWY and B groups, and Haemophilus influenzae type B. Their administration and booster schedules are detailed in various guidelines [100,101]. AIHA patients who may be candidate to splenectomy should be preferably vaccinated 2–4 weeks before administration of rituximab [114,115]. Uncertainty exists about antibiotic prophylaxis after splenectomy. Most guidelines advise it for the first 2–3 years, when the infectious risk is highest, or lifelong in highly comorbid patients [100,101,115]. However, the only evidence for antibiotic prophylaxis is derived from two old studies in pediatric sickle cell disease [116,117], while no studies are available in adults. More importantly, patients should be educated in recognizing infectious symptoms, starting broad-spectrum antibiotics, and promptly referring to hospital in case of persistent fever [100]. Regarding eculizumab, quadrivalent and group B meningococcal vaccines are recommended [118,119], and antibiotic prophylaxis should be instituted until vaccinations have been performed [102]. Clinical trials with complement inhibitors in AIHA, e.g., sutimlimab and pegcetacoplan, require vaccination against all encapsulated bacteria (NCT03347396, NCT03347422, NCT03500549).

Finally, non-live vaccines can be safely administered in adult patients with autoimmune inflammatory rheumatic diseases, whereas live-attenuated vaccines should be considered with caution [120]. The Food and Drug Aministration advises against live or live attenuated vaccines in patients receiving >10 mg/day of prednisone or a cumulative dose >700 mg in 3 months, recommending to defer them at least one month after steroid discontinuation [102].

## 6. Infections in AIHA Secondary to Other Diseases

AIHA can develop secondarily to several conditions (Table 5): PIDs [121,122,123], systemic autoimmune disorders [124,125,126], lymphoproliferative diseases [127,128], solid cancers, solid organ, and hematopoietic stem cell transplants (HSCT) [129,130,131]. All these conditions are characterized by an increased infectious risk, due to the intrinsic immunodeficiency and/or immunosuppressants and chemotherapy administered. AIHA requiring immunosuppressive therapy in these contexts may further increase the infectious risk. PIDs are characterized by an increased frequency of infections, mainly upper and lower respiratory tract ones [132,133]. Regarding autoimmune diseases, infections are recognized as the major cause of death in hospitalized patients with SLE and Sjogren’s syndrome [134,135,136]. Interestingly, Wang et al. demonstrated that AIHA is a risk factor for the development of bloodstream infections in SLE, probably due to the increased need of steroids [137]. The association of AIHA with non-Hodgkin lymphomas and CLL is well known [127], with the highest frequency in the latter (5–20%) [128]. Infections are described in up to 80% of CLL patients, cause about 60% of deaths [138], and are possibly related to hypogammaglobulinemia, abnormal T cell function, defective innate immunity, and specific therapies. Regarding transplants, the infectious risk is highly variable according to the organ transplanted, type of immunosuppressive therapy and time from transplantation. Bacterial infections are more frequent in the first weeks, secondarily to neutropenia for HSCT and surgical intervention/hospitalization for solid organs. Later, opportunistic and viral reactivations prevail, due to long-term immunosuppression [139,140,141]. AIHA is known to occur in up to 15% of HSCT after a median of 3–10 months from transplant, and is usually characterized by a severe/refractory course and fatal outcome. Risk factors for AIHA secondary to HSCT include use of unrelated donor and HLA-mismatch, occurrence of graft-versus-host disease, use of cord blood, age <15 years, CMV reactivation, alemtuzumab use, and non-malignant condition pre-HSCT [142]. As for lymphoproliferative disorders, it is difficult to establish the specific contribution of AIHA treatments to the occurrence of infections, since HSCT is already marked by a profound alteration of immune homeostasis.

## 7. AIHA Secondary to Infections

AIHA can be secondary to infections [143], particularly in children and with a prevalence of cold forms. Infectious agents can trigger AIHA through various mechanisms, including modification of erythrocyte membrane antigens, polyclonal B cell activation, innocent bystander and molecular mimicry [144,145]. Regarding viruses, parvovirus B19 [146] and hepatotropic viruses are the most frequently described [147,148]. A large population study including more than 120,000 HCV-infected American veterans concluded that the development of AIHA seems to be associated with HCV treatment with interferon [149]. Additionally, AIHA may complicate about 3% of infectious mononucleosis, with a typical onset within 1–2 weeks [150]. Paroxysmal cold hemoglobinuria, a rare form of AIHA caused by a biphasic hemolysin, more commonly occurring in children, is almost invariably preceded by a viral infection [151]. Regarding bacterial infections, Mycoplasma pneumoniae may be accompanied by severe AIHA, mainly cold but even warm forms [152]. It is worth mentioning cold AIHA secondary to *Mycobacterium tuberculosis* infection, with a reported efficacy of anti-tuberculosis treatment also on hemolytic anemia [153]. Moreover, case reports of AIHA have been described due to acute brucellosis [154]. Most recently, about 20 cases of AIHAs (both cold and warm forms) secondary to COVID-19 infection have been reported, with only one fatality [155]. Most of them recovered after first-line therapy with steroids +/− intravenous immunoglobulins [156,157], and some spontaneous remissions are also described [158].

## 8. Conclusions

**Figure 1:**
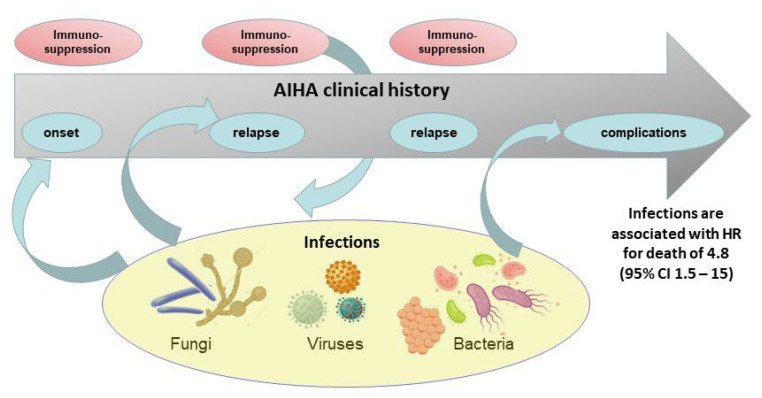
Relationships between autoimmune hemolytic anemia (AIHA) and infections during disease course. Infectious agents can be triggers for AIHA development at onset or at relapse (AIHA secondary to infections). On the other hand, immunosuppressive agents administered to treat AIHA expose the patient to infective events (infections secondary to AIHA), which are associated to disease complications and death.

The infectious burden in AIHA is considerable, consisting also in atypical and opportunistic infections, and representing a risk factor for morbidity and mortality (Figure 1). Infections are mainly associated with the load of therapy, with some peculiarity for certain treatments. Steroids encompass the widest spectrum of infections, with an underestimated risk, particularly for high doses and long-term administration, without clear indications for prophylaxis. Infections after splenectomy, mainly involving encapsulated bacteria, may be lowered by vaccination policies and antimicrobial prophylaxis/patient’s education. Rituximab is generally safe, although combination therapies deserve particular attention. Among the classic immunosuppressants, a higher infectious risk exists for cyclophosphamide. Trials with novel agents have identified specific drug-related infections and recommended preventive strategies, although long-term safety data are warranted. Finally, except for HBV reactivation and post-splenectomy vaccinations, preventive strategies for other infective agents still represent an unmet need in AIHA.

## Figures and Tables

**Table 1:** Infectious risk associated with autoimmune hemolytic anemia (AIHA) treatments.

Treatment	Main Warnings	References
Corticosteroids	- Infectious risk is dose-dependent - Also prolonged use of low-dose steroids is associated with atypical and opportunistic infections	[10,11,12]
Rituximab	- Safe as single agent - Risk of hepatitis B virus reactivation, if antiviral prophylaxis not instituted - Risk increases in chemotherapy-combined regimens or in the context of severe immunodepression (warning for PML)	[13,14,15,16,17]
Splenectomy	- Infections in 6–7% of AIHA patients - *Encapsulated bacteria* are the main pathogens isolated in OPSI, which can be fatal - Risk decreases with proper patient’s education and vaccinations	[18,19]
Classic immunosuppressive agents	- CTX, MMF, and AZA are associated with increased infectious risk by several pathogens - Cyclosporine seems safer than the abovementioned drugs	[20,21,22]
Complement inhibitors	- Increased risk of encapsulated bacterial infections	[23,24]
BCR pathway antagonists	- PI3Kδ inhibitors are associated to PJP - Fostamatinib (used in RA patients) has an increased infectious risk	[25,26]
Proteasome inhibitors	- Apparently safe in AIHA	[27,28]
FcRn antagonists	- Reported to be safe in ITP patients	[29,30]

PML: progressive multifocal leukoencephalopathy, AIHA: autoimmune hemolytic anemia, OPSI: overwhelming post-splenectomy infection, CTX: cyclophosphamide, MMF: mycophenolate mofetil, AZA: azathioprine, BCR: B-cell receptor, PI3Kδ: phosphoinositide 3-kinase delta, PJP: *Pneumocystis jirovecii* pneumonia, RA: rheumatoid arthritis, FcRn: neonatal Fc receptor, ITP: immune thrombocytopenia.

**Table 2:** Risk of hepatitis B virus (HBV) reactivation for different AIHA treatments according to viral status.

Drug	Viral Status	Risk Category
High-dose steroids *	HBsAg+HBsAg-/antiHBc+	high moderate
Moderate-dose steroids **	HBsAg+HBsAg-/antiHBc+	moderate low
Short-term low-dose steroids ***	irrelevant	low
Rituximab	irrelevant	high
Cyclosporine	irrelevant	moderate
Methotrexate	irrelevant	low
Azathioprine	irrelevant	low
Bortezomib	irrelevant	moderate

HBsAg: hepatitis B surface antigen, antiHBc: hepatitis B core antibodies, high risk: >10% rate of HBV reactivation, moderate risk: 1–10% rate of HBV reactivation, low risk: <1% rate of HBV reactivation, * >20 mg prednisone-equivalent dose/day, ** 10–20 mg prednisone-equivalent dose/day, *** <10 mg prednisone-equivalent dose/day over 4 weeks.

**Table 3:** Prevention strategies detailed per pathogen.

Pathogen	Screening Test	Risk Factors	Prophylaxis	References
HBV	HBsAg, antiHBs, antiHBc, antiHBe, HBeAg, HBV-DNA when indicated	Steroids Rituximab Immunosuppressors Bortezomib	- Lamivudine, entecavir, tenofovir or pre-emptive therapy according to EASL or AGA guidelines - HBV vaccination of seronegative patients	[93,94]
HCV	Anti-HCV (HCV-RNA if Ab positive)	Long-term steroids Rituximab	- No drugs approved for prophylaxis - Eradication therapy in HCV-RNA+	[9,95]
*Mycobacterium tuberculosis*	tuberculin skin test or serum interferon gamma release assays +/- chest X-ray	Long-term steroids	Isoniazid (or rifampicin) in latent TB, polichemotherapy in active TB	[96]
*Pneumocystis jirovecii*	No screening tests available	Steroids >10 mg/day + age >65 or pulmonary disease or therapy with rituximab/CTX	TMP-SMX (atovaquone, pentamidine, dapsone if not tolerated/contraindicated)	[9,97,98]
*Encapsulated bacteria*	No screening tests available	Splenectomy Complement inhibitors	- ACWY and B group meningococcal vaccines - 23-valent and 13-valent pneumococcal vaccines - Haemophilus influenzae type B vaccine	[23,99,100,101,102]

HBV: hepatitis B virus, HCV: hepatitis C virus, HBsAg: hepatits B surface antigen, antiHBs: hepatits B surface antibodies, antiHBc: hepatitis B core antibodies, antiHBe: hepatitis B e-antibodies, HBeAg: hepatitis B e-antigen, HCV: hepatitis C virus, Ab: antibodies, EASL: European Association for the Study of the Liver, AGA: American Gastroenterological Association, TB: tuberculosis, CTX: cyclophosphamide, TMP-SMX: trimethoprim-sulfamethoxazole.

**Table 4:** HBV reactivation prophylactic strategies according to risk category.

Risk Category	Preventive Strategy Recommended	
	EASL Guidelines (2017)	AGA Guidelines (2015)
High (>10%)	Entecavir or tenofovir if HBsAg+ Lamivudine if HBsAg-/antiHBc+	Entecavir or tenofovir
Moderate (1–10%)	Pre-emptive therapy	Entecavir or tenofovir
Low (<1%)	Pre-emptive therapy	Pre-emptive therapy

EASL: European Association for the Study of the Liver, AGA: American Gastroenterological Association, HBsAg: hepatitis B surface antigen, antiHBc: hepatitis B core antibodies.

**Table 5:** Infectious risk in conditions associated with AIHA.

Condition	Frequency of AIHA	Characteristics of Infections	References
Primary immunodeficiencies			
Autoimmune lymphoproliferative syndrome	29%	Up to 30% of patients with bacterial infections related to neutropenia and splenectomy	[121]
IgA deficiency	15%	40–90% of patients experience recurrent respiratory infections, cases of Giardia lamblia infections	[122,133]
Common variable immundeficiency	2–5%	50% of subjects with upper respiratory tract infections, 50% pneumonia, 40% diarrhea, 10% VZV reactivation	[123,132]
Autoimmune diseases			
Systemic lupus erythematosus	3–14%	43.1/1000 patients/year incidence of opportunistic infections; Infection is a major cause of death	[124,134,135]
Sjogren syndrome	2–3%	24.1/1000 patients/year incidence of opportunistic infections; infections are one of the main causes of death	[125,134]
Inflammatory bowel diseases	0.05%	Up to 30% of treated patients experienced infections	[126,136]
Neoplasms			
Chronic lymphocytic leukemia	5–20%	Frequency of drug specific infections: 57% FC; 40% FCR (mainly bacterial/opportunistic); 27% alemtuzumab (mainly CMV and fungi); 12–45% anti-CD20 (HBV, CMV, HSV, VZV, PML); 13–50% with novel drugs (ibrutinib, skin, respiratory tract, UTIs; idelalisib, PJP, CMV; venetoclax, bacterial infections)	[128,138]
Transplants			
Hematopoietic stem cell transplant	10–15%	7–50% bacteriemia; 11–24% airways, GI tract, skin and soft tissue infection; 4–20% UTIs; 1–5% PJP (without prophylaxis); 23% invasive aspergillosis; 30–50% CMV reactivation	[131,139]
Solid organs transplant	2–3%	Frequent bacterial infections in the first month after transplant; 30–97% CMV reactivation; 2% Candida; 1–2% invasive aspergillosis; 1–2% cryptococcosis	[130,140,141]

VZV: varicella zoster virus, FC: fludarabine-cyclophosphamide, FCR: fludarabine-cyclophosphamide-rituximab, HBV: hepatitis B virus, CMV: cytomegalovirus, HSV: herpes simplex virus, PML: progressive multifocal leukoencephalopathy, UTIs: urinary tract infections, PJP: pneumocystis jirovecii pneumonia, GI: gastrointestinal.
